# Loss of Parkin Results in Altered Muscle Stem Cell Differentiation during Regeneration

**DOI:** 10.3390/ijms21218007

**Published:** 2020-10-28

**Authors:** Marcos V. Esteca, Matheus B. Severino, João G. Silvestre, Gustavo Palmeira dos Santos, Letícia Tamborlin, Augusto D. Luchessi, Anselmo S. Moriscot, Åsa B. Gustafsson, Igor L. Baptista

**Affiliations:** 1 Laboratory of Cell and Tissue Biology, School of Applied Sciences, University of Campinas, 13484-350 Limeira, Brazil; marcos.esteca@gmail.com (M.V.E.); matheusbr.severino@gmail.com (M.B.S.); gustavopalmeira7@gmail.com (G.P.d.S.); 2 Department of Anatomy, Institute of Biomedical Sciences, University of São Paulo, 05508-900 São Paulo, Brazil; jgsilvestre@gmail.com (J.G.S.); moriscotanselmo@gmail.com (A.S.M.); 3 Laboratory of Biotechnology, School of Applied Sciences, University of Campinas, 13484-350 Limeira, Brazil; leticia_tamborlin@hotmail.com (L.T.); augusto.luchessi@gmail.com (A.D.L.); 4 Institute of Biosciences, São Paulo State University, 13506-900 Rio Claro, Brazil; 5 Skaggs School of Pharmacy and Pharmacological Sciences, University of California San Diego, La Jolla, CA 92093-0021, USA; abgustafsson@health.ucsd.edu

**Keywords:** satellite cells, differentiation, mitochondria, mitophagy

## Abstract

The high capacity of the skeletal muscle to regenerate is due to the presence of muscle stem cells (MuSCs, or satellite cells). The E3 ubiquitin ligase Parkin is a key regulator of mitophagy and is recruited to mitochondria during differentiation of mouse myoblast cell line. However, the function of mitophagy during regeneration has not been investigated in vivo. Here, we have utilized Parkin deficient (Parkin^–/–^) mice to investigate the role of Parkin in skeletal muscle regeneration. We found a persistent deficiency in skeletal muscle regeneration in Parkin^–/–^ mice after cardiotoxin (CTX) injury with increased area of fibrosis and decreased cross-sectional area (CSA) of myofibres post-injury. There was also a significant modulation of MuSCs differentiation and mitophagic markers, with altered mitochondrial proteins during skeletal muscle regeneration in Parkin^–/–^ mice. Our data suggest that Parkin-mediated mitophagy plays a key role in skeletal muscle regeneration and is necessary for MuSCs differentiation.

## 1. Introduction

Skeletal muscle has high capacity to regenerate after injury because of the presence of MuSCs, also known as satellite cells, between the basal membrane and the sarcolemma [1]. In a healthy adult skeletal muscle, MuSCs remain in their quiescent state due to expression of paired-box transcription factor 7 (Pax7) [2]. This transcription factor has been reported to be critical for maintaining the pool of MuSCs [1,2]. Muscle injury leads to activation of MuSCs and upregulation of various cell cycle factors to initiate proliferation [3]. The activation of MuSCs, also called myoblasts at this stage, is the first step in the repair process and occurs during the inflammatory phase, within 1–3 days post-injury (dpi). After the inflammatory phase, the regenerative phase is initiated with a portion of the MuSCs differentiating into myocytes [4]. Fusion of myoblasts results in formation of multinucleated myotubes.The differentiation process is characterized by the upregulation of Myogenin, which is one of the myogenic regulatory factors responsible for inducing cell cycle exit, terminal differentiation, and expression of factors that enable the fusion of MuSCs [5]. During the regenerative phase, cells with centralized nuclei emerge from differentiated myocytes marked by the expression of Myh3 (embryonic myosin). During the growth and maturation process of myofibres around 7dpi, there is a decrease in Myh3 expression [6]. The maturing myofibres also start expressing different adult isoforms of myosin. During this phase, the extracellular matrix and angiogenesis are restored in the injured area.

The signaling pathways involved in regulating the differentiation process in MuSCs are complex and under intense investigation. Recent studies have identified that mitochondrial autophagy or mitophagy is activated during differentiation [7,8,9]. Mitophagy is the selective removal of damaged or unwanted mitochondria by autophagosomes [10]. Activation of mitophagy has been reported to be a requirement for mitochondrial network remodeling during the differentiation and fusion of C2C12 myotubes [7,8]. Interestingly, Baechler et al. demonstrated that impaired mitophagy results in decreased levels of Myogenin in C2C12 cells, showing a direct relation between mitophagy and myogenic program [7]. One important player on mitophagy, the E3 ubiquitin ligase Parkin has been shown to initiate the mitophagic process in several cell types by mediating the ubiquitination of mitochondrial outer membrane proteins (e.g., VDACs and Toms), recruiting the autophagic machinery to dysfunctional mitochondria [11,12]. In skeletal muscle, Parkin has been shown to be essential for mitochondrial quality control, and its absence causes atrophy and loss of muscle function [13]. However, it is still unclear whether Parkin functions in skeletal muscle repair after injury. In this study, we have evaluated the role of Parkin in the regenerative process of skeletal muscle. We focused on Parkin effects on autophagy and mitochondrial reorganization, and the downstream consequence of Parkin-deficiency on the differentiation capacity of MuSCs.

## 2. Results

### 2.1. Parkin Is Expressed in MuSCs during Regeneration In Vivo and in Myoblast in Culture

**Figure 1:**
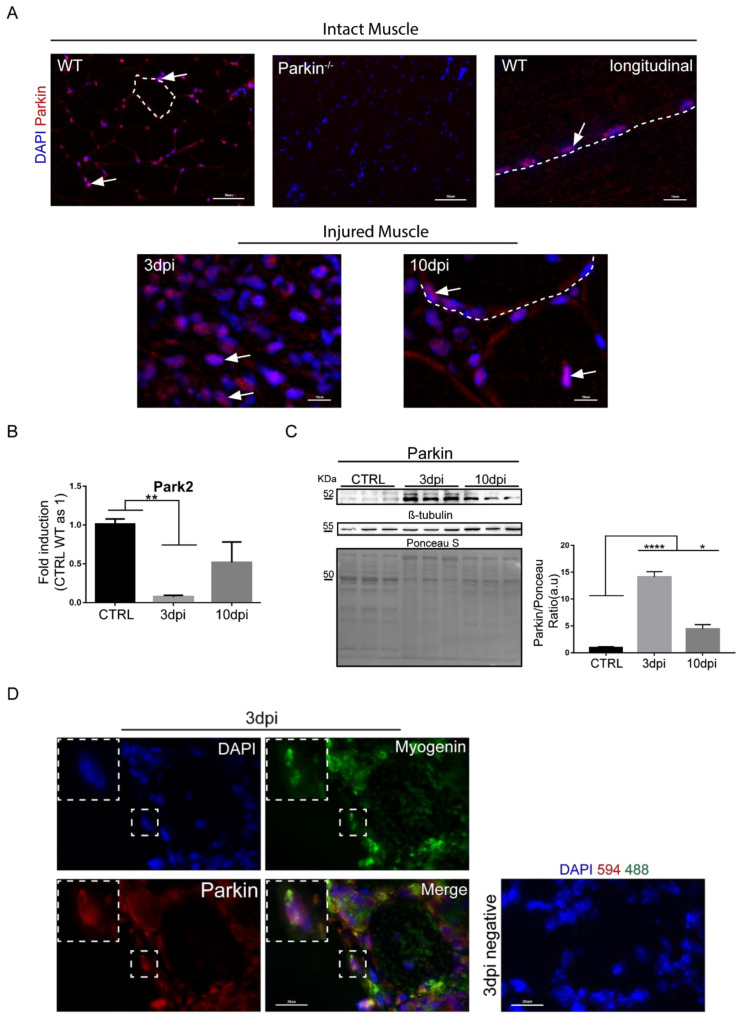
Parkin expression and localization during muscle regeneration. (**A**) Immunofluorescence staining for Parkin (red) and nuclei (blue) in the intact and injured TA muscle from WT mice in CTRL and at 3 and 10 dpi. The intact muscle of Parkin^–/–^ mice was used to confirm the specificity of the antibody staining. The arrows indicate Parkin’s location near the nuclei and the dotted areas delineate myofibers. The images were acquired with 40× and 100× objectives. Arrows indicate the location of Parkin near the nuclei. The myofibers are marked by dotted area. Bars in upper panel represent 50 µm; bars in lower panel represent 10 µm. (**B**) Park2 relative mRNA expression (RT-PCR) in TA muscle from WT mice at 3 and 10dpi compared with CTRL. Data represent means ± SD (One-Way ANOVA: ** p *<* *0.01 vs Control*; *n* = 5). (**C**) Western Blot analysis of Parkin in TA muscle from WT mice in CTRL at 3 and 10dpi. Densitometry analysis of proteins. Ponceau S staining was used to normalize for loading. β-tubulin was modulated in this model and Ponceau was used as loading control. Data represent means ± SEM (One-way ANOVA: * *p* < 0.05; **** *p* < 0.0001; *n* = 6). (**D**) Immunofluorescence of Parkin (red), Myogenin (green), and nuclei (blue) in TA muscle cryosections from WT mice at 3dpi. Right panel is a representative image of negative controls (split channels are presented in Figure S1E). The images were acquired with a 100× objective.

To investigate the importance of Parkin in MuSCs function, we induced skeletal muscle regeneration by injecting mice with a single dose of CTX into the tibialis anterior muscle in mice. CTX is known to act as a myotoxic agent which causes mitochondrial damage and disruption of myofiber inducing muscle injury [14]. We analyzed regeneration of muscle tissue at various time points (3, 10, and 21 days) post-injury. First, we evaluated cellular localization and expression levels of Parkin in TA muscle at baseline and after CTX injury. Immunostaining of TA muscle sections showed cytoplasmic and perinuclear localization for Parkin in mononuclear cells, in the non-injured control (CTRL) group (Figure 1A). Three days post-injury, we noted that mononuclear cells showed increased Parkin staining compared to other cells in the muscle tissue, suggesting that Parkin is selectively increased in these cells in response to injury. Ten days post-injury, Parkin was found both in mononuclear cells and in newly assembled myofibre, as histologically characterized by centralized nuclei (Figure 1A). We also noted that Parkin staining was more intense near the centralized myonuclei (Figure 1A). Analysis of Parkin transcript levels in TA muscle by real-time PCR revealed a significant decrease in *Park2* mRNA at 3dpi (Figure 1B). Unexpectedly, we found a significant increase in Parkin protein levels at both 3 and 10 days post-injury in TA muscle (Figure 1C). Many standard loading controls are also altered during regeneration and we confirmed that these are also modulated by CTX injection (Figure S1A–D). Therefore, we used Ponceau S staining to normalize for loading in our western blot experiments. Next, we analyzed whether the mononuclear cells that were positive for Parkin were also committed to the myogenic program. Immunostaining for Parkin and the myogenic marker Myogenin [5] in TA muscle confirmed that the cells Myogenin^+^ were also Parkin^+^ at 3 days post-injury (Figure 1D and negative controls showed in Figure S1E). These findings confirm that Parkin is expressed in MuSCs during the inflammatory phase and formation of newly assembled myofibre during the regenerative process.

**Figure 2:**
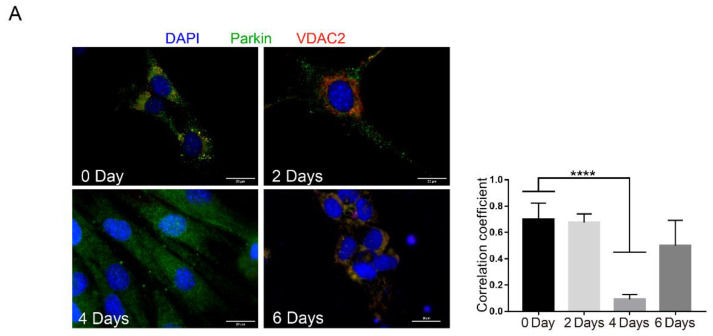

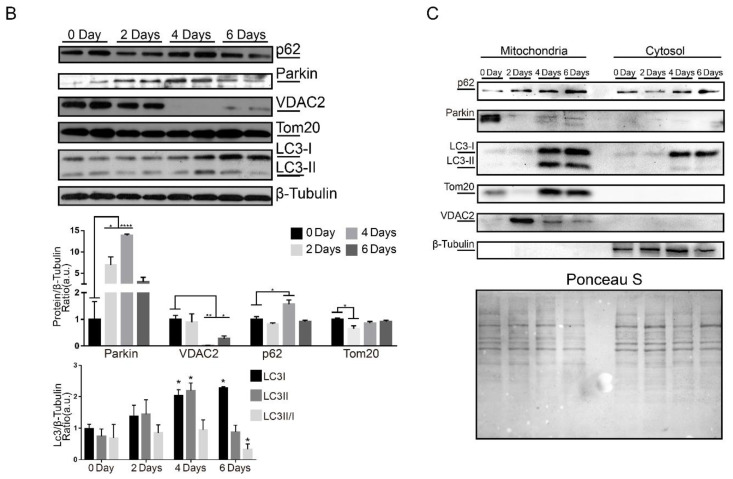
Parkin and mitophagy related proteins expression during C2C12 differentiation. (**A**) Immunofluorescence of Parkin (green), VDAC2 (red), and nuclei (blue) from C2C12 pre-differentiation (0 days) and at 2, 4, and 6 days post-differentiation. Bar graph shows each Pearson’s Correlation Coefficient. Data represent means ± SD (One-way ANOVA: **** *p* < 0.0001; *n* = 5). The images were acquired with a 100× objective. Bars represent 20 µm. (**B**) Western blot analysis and densitometry of p62, Parkin, VDAC2, Tom20, LC3-I and -II, and β-Tubulin from C2C12 pre-differentiation (0 days) and after 2, 4, and 6 days after differentiation. Data represent means ± SEM (One-way ANOVA: * *p* < 0.05, ** *p* < 0.01, **** *p* < 0.0001; *n* = 5). (**C**) Western blot analysis of p62, Parkin, LC3-I and -II, VDAC2, Tom20, and β-Tubulin in mitochondrial fractions from C2C12 pre-differentiation (0 days) and at 2, 4, and 6 days post-differentiation (*n* = 5). Ponceau S was used as a loading control.

To further analyze Parkin dynamics during myoblast differentiation, we evaluated Parkin localization in C2C12 cells (Figure 2 and Figure S1F). Using immunofluorescence, we observed high levels of co-localization between Parkin and the mitochondrial protein VDAC2 at baseline followed by a significant decrease in co-localization after 4 days of differentiation (Figure 2A). It is important to note that there is a significant decrease in VDAC2 at this time point (Figure 2B). Considering the importance of Parkin in regulating mitophagy, we analyzed levels of two different mitochondrial proteins, VDAC2 and Tom20, and autophagy proteins LC3 and p62. Western blot demonstrated that Parkin levels increased while VDAC2 decreased 4 days after differentiation. Tom20 was modestly but significantly reduced at 2 days and restored to normal levels by Day 4 (Figure 2B). We also observed LC3I accumulation after 4 and 6 days of differentiation but no significant changes in p62 levels at these time points (Figure 2B). Similarly, we analyzed the mitochondrial recruitment of Parkin and autophagy markers by western blot of mitochondrial fractions. As shown in Figure 2C shows higher levels of Parkin at the mitochondria at day 0 (pre-differentiation) and a reduction at 2, 4 and 6 days after initiation of differentiation. We also observed increased levels of mitochondrial LC3 after 4 and 6 days of differentiation, with increased levels of Tom20 (Figure 2C).

We used VDAC2 as second loading control and, curiously, noted an increased level of VDAC2 at 2 days of differentiation. Mitochondrial p62 levels remained the same during the differentiation process in C2C12 cells. Our cell culture results showed the recruitment of Parkin to mitochondria and regulation of mitochondrial markers during the differentiation. It is important to note that the alterations in Tom20 and VDAC2 levels suggest differential mitochondrial function and demand at various time points during the differentiation process.

### 2.2. Parkin Knockout Mice Show Reduced Ability to Undergo Skeletal Muscle Repair after Injury

**Figure 3:**
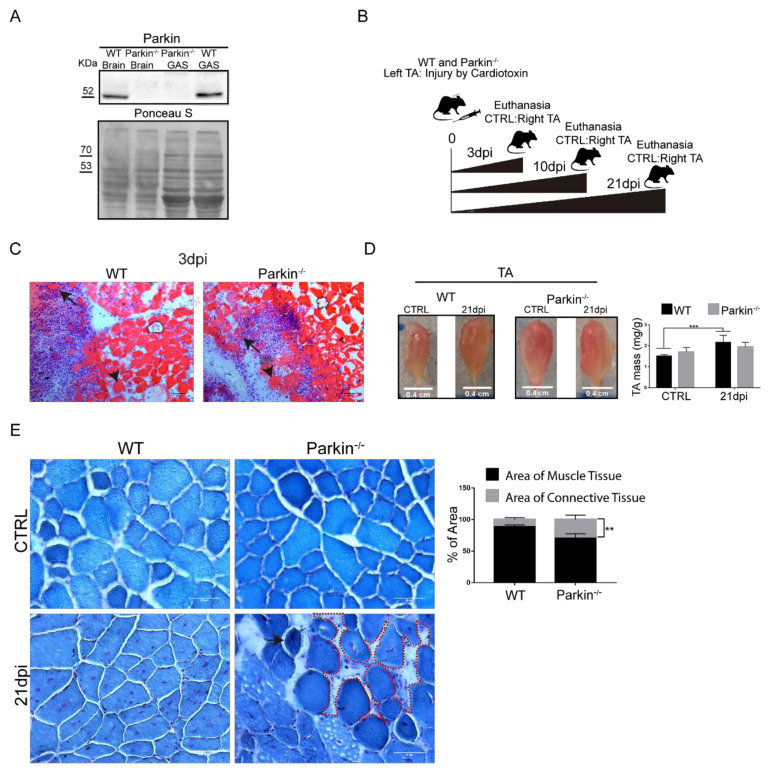
Absence of Parkin impairs skeletal muscle plasticity during the regenerative process. (**A**) Western Blot of Parkin in Gastrocnemius (GAS) muscle and brain from Parkin^−/−^ mice. (**B**) Experimental design for evaluating the regenerative process in mice. (**C**) Confirmation of injury during inflammatory phase by hematoxylin and eosin staining of TA muscle from WT and Parkin^−/−^ mice at 3dpi. Black arrows denote necrotic fibers, arrow heads mark hypercontracted fibers, and dotted areas outline ghost fibers. The images were acquired with a 20× objective. Bars represent 50 µm. (**D**) Representative images of CTRL and 21dpi of TA muscle from WT and Parkin^−/−^ mice. Graph show TA weight (mg)/Body weight (g) ratio. Data represent means ± SD (two-way ANOVA: Interaction *p* = 0.0285; Row Factor, injury Factor, *p* < 0.0001; Column Factor, genotype Factor, *p* = 0.9023; Bonferroni’s multiple comparisons test *** *p* < 0.01; *n* = 5). (**E**) Gomori staining of cryosections of CTRL and at 21dpi TA muscle from WT and Parkin^−/−^ mice. The dotted area indicates accumulation of connective tissue and black arrow indicate hypercontracted fiber in Parkin^−/−^ mice. Bar graphs show % of area occupied by muscle tissue and connective tissue in TA muscle from WT and Parkin^−/−^ mice at 21dpi. Data represent means ± SD *t*-test: ** *p* < 0.01; *n* = 5). The images were acquired with a 40× objective. Bars represent 50 µm.

To further investigate the role of Parkin in skeletal muscle repair after injury, Parkin^–/–^ mice were subjected to CTX injection in the TA muscle to induce injury (Figure 3A,B). First, we observed clear regeneration phenotype in both WT and Parkin^–/–^ mice including ghost fibers (dotted area), necrotic (black arrow), and hypercontracted fibers (arrow head) at 3dpi (Figure 3C). We observed an increase in TA muscle mass 21 days post-injury in WT mice. In contrast, Parkin^–/–^ mice showed no increase in TA muscle mass (Figure 3D). We also investigated whether lack of Parkin affected the tissue remodeling process. Using Gomori staining of 21 dpi, we observed reminiscent hypercontracted fibers (black arrow) and a larger area occupied by connective tissue in muscles of Parkin^–/–^ mice (Figure 3E and Figure S2). These results suggest that Parkin-deficiency leads to persistent injury possible due to reduced repair or regenerative capacity.

### 2.3. Parkin Deficiency Leads to Impaired Assembly of Myofibres after Skeletal Muscle Injury

**Figure 4:**
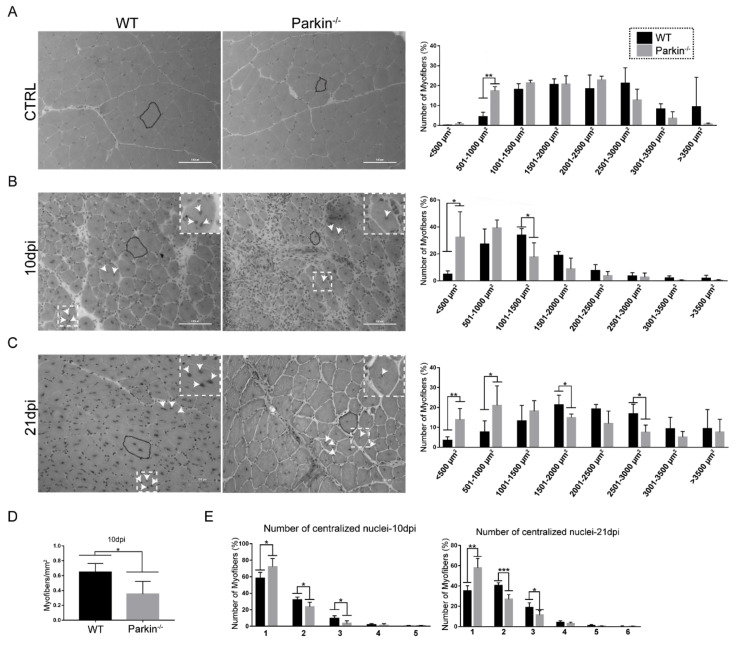
Loss of Parkinimpairs assembling of myofibres and growth of myofibers post-injury. (**A**) Hematoxylin and eosin staining in TA muscle cryosections from WT mice and Parkin^−/−^ mice in CTRL. The dotted areas demonstrate how the myofibers were circled for CSA analysis. Respective graph of CSA distribution profile in TA muscle myofibers. Data represent means ± SD (*t*-test: ** *p* < 0.01; *n* = 5). The images were acquired with a 20× objective. Bars represent 100 µm. (**B**) Hematoxylin and eosin staining of TA muscle cryosections from WT and Parkin^−/−^ mice at 10dpi. The black dotted areas demonstrate how myofibers were circled for CSA analysis, the white arrows indicate the centralized nuclei. Respective graph of CSA distribution profile of TA muscle myofibers. Data represent means ± SD (*t*-test: * *p* < 0.05; *n* = 5). The images were acquired with a 20× objective. Bars represent 100 µm. (**C**)Hematoxylin and eosin staining of TA muscle cryosections from WT and Parkin^−/−^ mice at 21dpi. The black dotted areas show how the myofibers were circled for CSA analysis, the white arrows indicate the centralized nuclei. Respective graph of CSA distribution profile of TA muscle myofibers. (*t*-test: * *p* < 0.05; ** *p* < 0.01; *n* = 5). The images were acquired with a 20× objective. Bars represent 100 µm. (**D**) Quantitation of myofibers with centralized nucleus per mm^2^ in TA muscle from WT and Parkin^−/−^ mice at 10dpi. Data represent means ± SD (*t*-test: * *p* < 0.05; *n* = 5). (**E**) Quantitation of centralized nuclei per cell in TA muscle myofibers from WT and Parkin^−/−^ mice at 10 and 21dpi. Data represent means ± SD (*t*-test: * *p* < 0.05; ** *p* < 0.01 and *** *p* < 0.001; *n* = 5).

Next, we investigated the ability of MuSCs to form new myofibres and to increase their size in absence of Parkin. First, we analyzed the CSA of myofibres in TA muscle from WT and Parkin^–/–^ mice at baseline (CTRL). We found that Parkin^–/–^ mice had a larger number of myofibers between 501–1000 μm^2^ in CSA (Figure 4A). At 10 dpi, muscles of Parkin^–/–^ mice demonstrated higher frequency of myofibres with CSA between <500 μm^2^ and lower frequency of 1001–1500 μm^2^ compared with WT mice (Figure 4B). The TA muscle of Parkin^–/–^ mice at 10dpi also showed increased number of myofibres, with only one centralized nucleus, and reduced number of myofibreswith two or three centralized nuclei (Figure 4E). We also observed a reduced number of myofibres per area in skeletal muscles of Parkin^–/–^ mice at 10dpi (Figure 4D). We obtained similar results in the Parkin^−/−^ at 21dpi (Figure 4C,E). These results suggest that myofibre growth is reduced in absence of Parkin.

### 2.4. Parkin^−/−^ MuSCs Display Reduced Differentiation Capacity and Increased Proliferation

**Figure 5:**
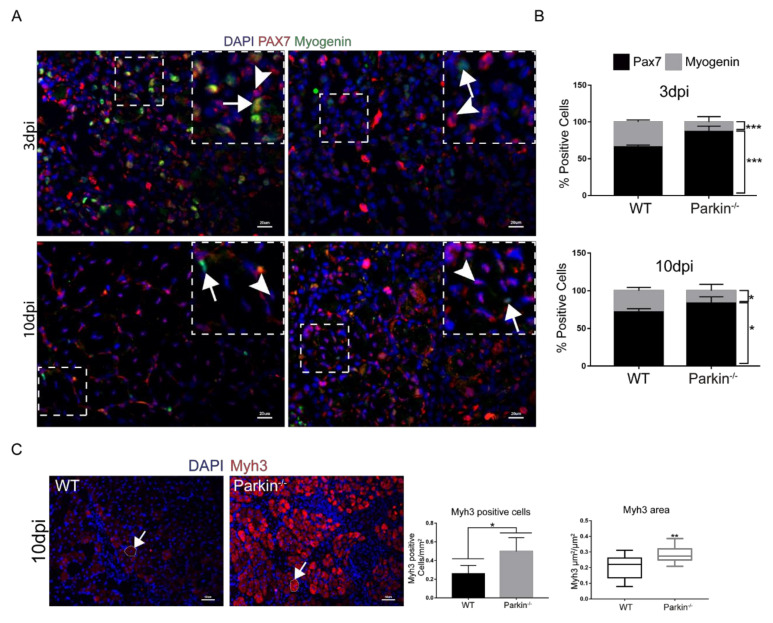

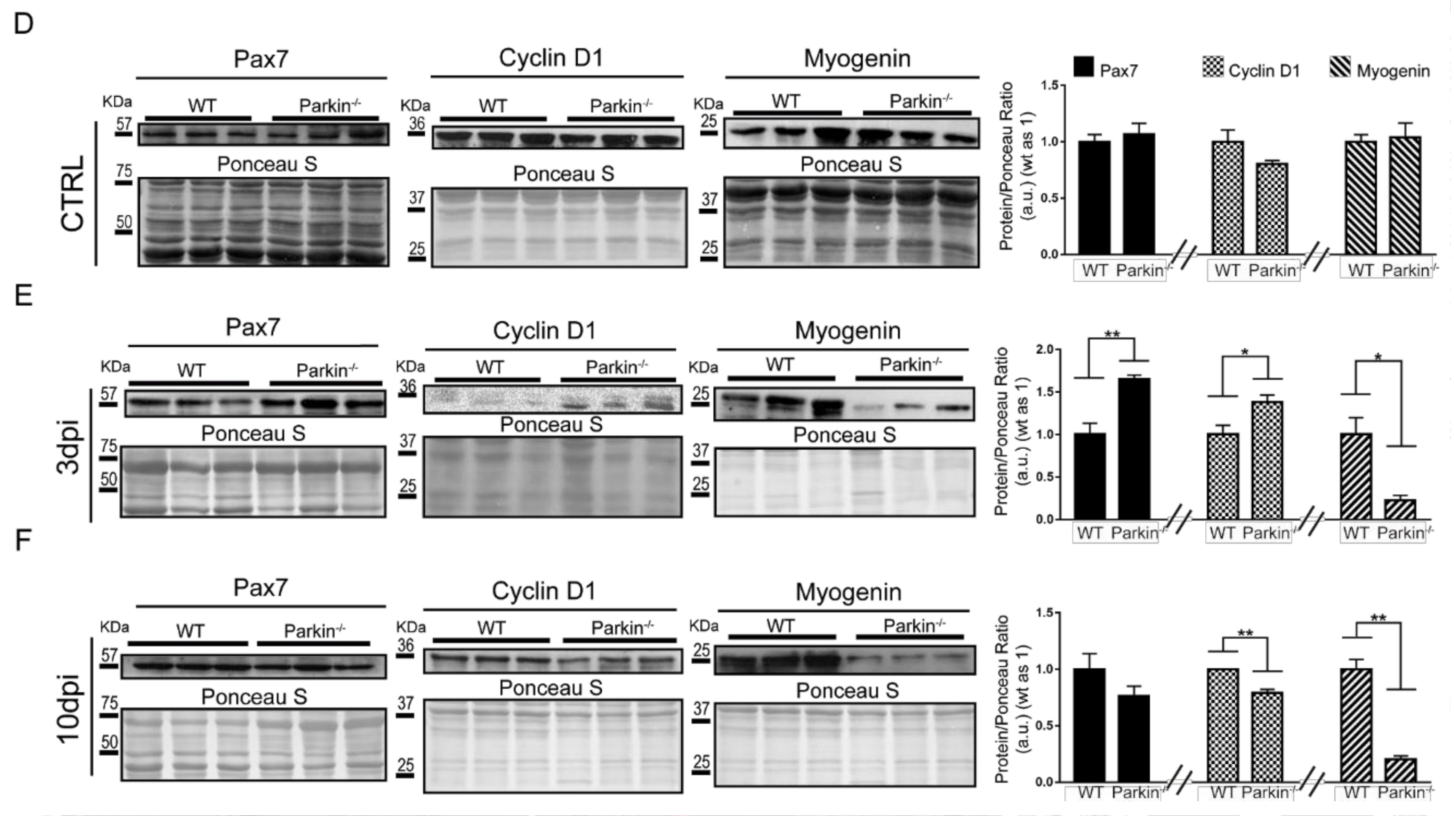
Absence of Parkin results in commitment of MuSCs to cell cycle and reduced Myogenin expression. (**A**) Immunofluorescence of Pax7 (red), Myogenin (green), and DAPI (blue) in TA muscle cryosections from WT and Parkin^−/−^ mice at 3 and 10 dpi. White arrowheads indicate Pax7^+^ cells and white arrows indicate Myogenin^+^ cells. The split channels are presented in Figure S2. The images were acquired with a 40× and 20× objectives respectively. Bars represent 20 µm. (**B**) % of Pax7^+^ and Myogenin^+^ cell in TA muscle from WT and Parkin^−/−^ mice at 3 and 10dpi. Data represent means ± SD (*t*-test: * *p* < 0.05; *** *p* < 0.01; *n* = 5). (**C**) Immunofluorescence of Myh3 (red) and DAPI (blue) of TA muscle cryosections from WT and Parkin^−/−^ mice at 10dpi. The graphs represent Myh3 positive cells by area and total area occupied by Myh3 cells. The myofibers are marked by dotted areas. Data represent means ± SD (*t*-test: * *p* < 0.05; ** *p* < 0.01; *n* = 5). The images were acquired with a 20× objective. Bars represent 50 µm. (**D**) Western blot and densitometry analysis of Pax7, Cyclin D1, and Myogenin levels in TA muscle from WT and Parkin^−/−^ mice in CTRL. Ponceau S staining was used to normalize for loading. Data represent means ± SEM (*n* = 6). (**E**) Western blot and densitometry analysis of Pax7, Cyclin D1, and Myogenin levels in TA muscle from WT and Parkin^−/−^ mice at 3dpi. Ponceau S staining was used to normalize for loading. Data represent means ± SEM (*t*-test: * *p* < 0.05; ** *p* < 0.01; *n* = 6). (**F**) Western blot and densitometry analysis of Pax7, Cyclin D1, and Myogenin levels in TA muscle from WT and Parkin^−/−^ mice at 10dpi. Ponceau S staining was used to normalize for loading. Data represent means ± SEM (*t*-test: ** *p* < 0.01; *n* = 6).

To investigate the ability of Parkin^−/−^ MuSCs to differentiate and express myogenic markers, we characterized MuSC niches in TA muscle at 3dpi and 10dpi in WT and Parkin^−/−^ mice by immunofluorescence. We used Pax7 as a marker for undifferentiated MuSCswith proliferative potential, Cyclin D1 as a cell cycle marker (G1-S), and Myogenin as a marker of MuSCs committed to differentiation. Assessment of Pax7^+^ and Myogenin^+^ cells demonstrated that Parkin^−/−^ mice had higher percentage of Pax7^+^ MuSCs and lower percentage of Myogenin^+^ MuSCs at both 3 and 10 dpi compared to WT MuSCs (Figure 5A,B and split channels are presented in Figure S4A). Additionally, we used neural cell adhesion molecule (NCAM), a protein crucial for cell-to-cell contact and fusion initiation [15], as an initial marker of compromised MuSCs function. Unexpectedly, Parkin^−/−^ mice showed a higher content of NCAM^+^ MuSCs in TA muscle than WT mice in 3dpi (Figure S3A,B). 

To compare the maturation status of myofibre between WT and Parkin^−/−^ mice, we stained muscle tissue sections with anti-Myh3 (developmental myosin) and evaluated the number of positive cells by immunofluorescence. Interestingly, Parkin^−/−^ mice showed a significantly higher number of Myh3^+^ cells and incresead ocuppied area of Myh3 in skeletal muscle than WT mice at 10dpi (Figure 5C,D). Next, we analyzed the protein levels of Pax7, Myogenin, and Cyclin D1 but their levels were not significantly different in WT and Parkin^−/−^ TA muscle at baseline (Figure 5D). Three days post-injury, we found a significantly higher protein level of Pax7 and Cyclin D1 in skeletal muscle of Parkin^−/−^ mice (Figure 5E), while Myogenin were reduced at both 3 and 10dpi (Figure 5E,F). Altogether, these data show that Parkin-deficient MuSCs retain their proliferative potential during the regenerative process and are able to engage in differentiation. However, a defect exists in the final steps of differentiation which results in late maturation of myofiber, reduced myofibre size and decreased regenerative capacity in Parkin^−/−^ mice.

### 2.5. Parkin Deficiency Alters Mitochondrial Protein Levels during Skeletal Muscle Regeneration

**Figure 6:**
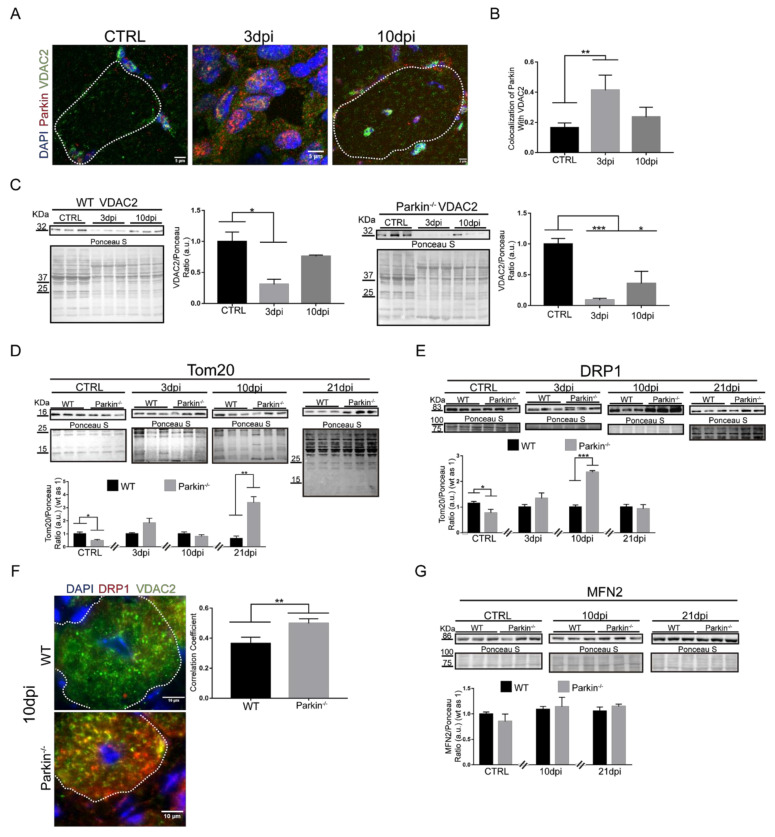

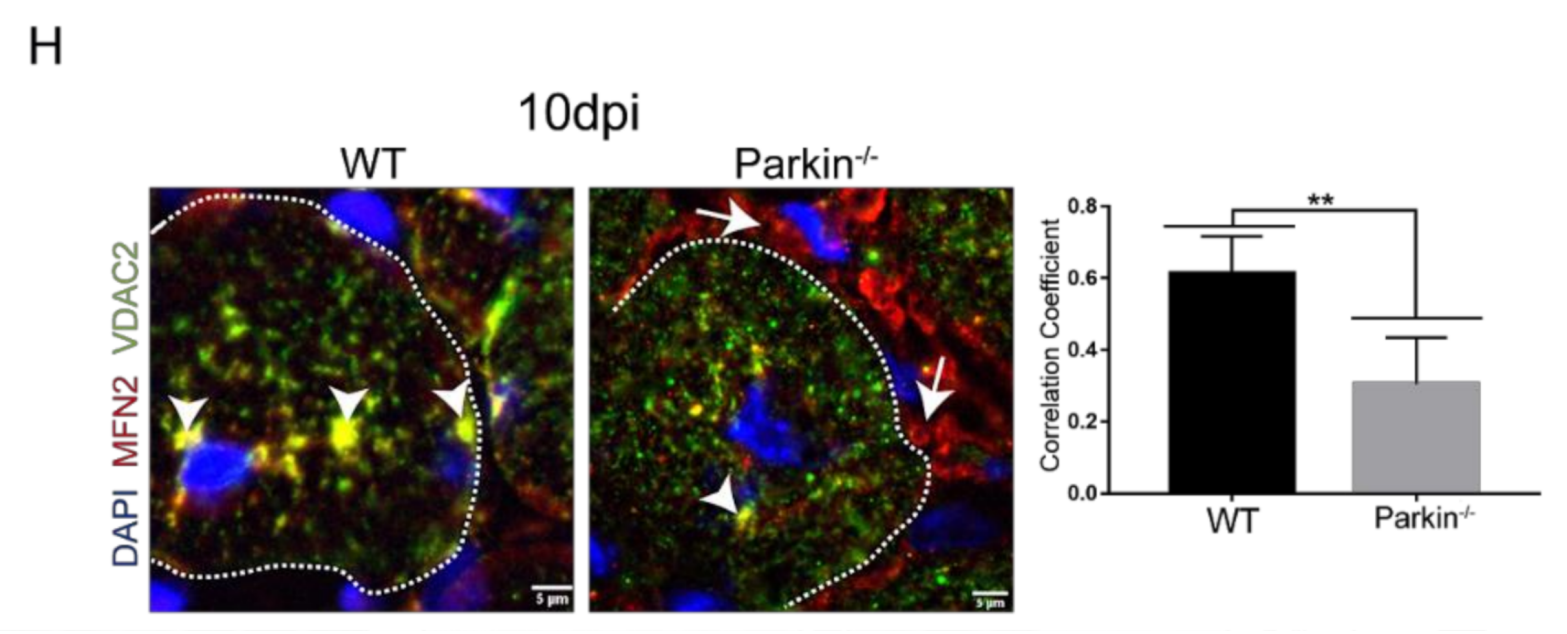
Parkin^−/−^ mice shows altered mitochondrial dynamics during skeletal muscle regeneration. (**A**) Immunofluorescence confocal images of Parkin (red), VDAC2 (green), and DAPI (blue) in TA muscle cryosections from in CTRL and 3 and 10dpi WT mice. The myofibers are labeled by dotted area. The images were acquired with a 63× objective. Bars represent 5 µm. (**B**) Colocalization of Parkin with VDAC2. Data represent means ± SD (*t*-test: ** *p* < 0.01; *n* = 5). (**C**) Western blot and densitometry analysis of VDAC2 levels during the regenerative process in CTRL, 3dpi and 10dpi TA muscle from WT and Parkin^−/−^ mice. Comparisons were made with the TA muscle in CTRL of each genotype. Ponceau S staining was used to normalize for loading. Data represent means ± SEM (One-way ANOVA: * *p* < 0.05; *** *p* < 0.001; *n* = 5). (**D**) Western blot and densitometry analysis of Tom20 levels during the regenerative process in CTRL, 3, 10 and 21dpi TA muscle from WT and Parkin^−/−^ mice. Ponceau S staining was used to normalize for loading. Data represent means ± SEM (*t*-test: * *p* < 0.05; ** *p* < 0.01; *n* = 5). (**E**) Western blot and densitometry analysis of DRP1 levels during the regenerative process in CTRL, 3dpi, 10dpi and 21dpi TA muscle from WT and Parkin^–/–^ mice. Ponceau S staining was used to normalize for loading. Data represent means ± SEM (*t*-test: * *p* < 0.05; *** *p* < 0.01; *n* = 5). (**F**) Immunofluorescence analysis of DRP1 (red), VDAC2 (green), and DAPI (blue) in TA muscle from WT and Parkin^−/−^ mice at 10dpi. Dotted areas mark the myofiber. Bar graph of Pearson’s Correlation Coefficient. Data represent means ± SD (*t*-test: ** *p* < 0.05; *n* = 4). The images were acquired with a 100× objective and a detail of the image is being evidenced for a better visualization. Bars represent 10 µm. (**G**) Western blot and densitometry analysis of MFN2 levels during the regenerative process in CTRL, 10 and 21dpi TA muscle from WT and Parkin^−/−^ mice. Ponceau S staining was used to normalize for loading. Data represent means ± SEM. (**H**) Immunofluorescence of MFN2 (red), VDAC2 (green), and DAPI (blue) in TA muscle cryosections from WT and Parkin^−/−^ mice at 10dpi. Dotted areas outline myofiber, white arrows indicate VDAC2/MFN2 co-localization and white arrows head indicate MFN2 staining at MuSCs. Bar graph shows Pearson’s Correlation Coefficient. Data represent means ± SD (*t*-test: ** *p* < 0.05; *n* = 5). The images were acquired with a 100× objective. Bars represent 5 µm.

It has previously been demonstrated that cardiotoxin-mediated skeletal muscle injury involves mitochondrial dysfunction and that these mitochondria must be eliminated by mitophagy to prevent cell death [14,16]. Therefore, we analyzed VDAC2 colocalization with Parkin during the regeneration process in TA skeletal muscle. We found high levels of colocalization between VDAC2 and Parkin at 3dpi the skeletal muscle of WT mice (Figure 6A,B). We can assume that labeling is present in MuSCs since ~60% of the cells at that time are myogenic (positive for Pax7 or Myogenin—Figure S3C). Interestingly, although we found no significant differences in the colocalization at 10dpi, we noted the presence of both Parkin and VDAC2 in the periphery and around the centralized nuclei of myofibres (Figure 6A). Analysis of VDAC2 protein levels during muscle regeneration demonstrated a decrease in the protein levels 3dpi in both WT and Parkin^–/–^ muscle (Figure 6C). At 10dpi, VDAC2 levels were restored to baseline levels in WT muscle but remained reduced in Parkin^−/−^ muscle (Figure 6C). To directly compare protein levels in WT and Parkin^−/−^, we performed western blot analysis at each time point and these results confirmed the reduction of VDAC2 in Parkin^−/−^ muscle (Figure S4).

Since we observed differential protein expression of VDAC2 (increased) and Tom20 (decreased) during C2C12 differentiation, we investigated whether Tom20 levels were altered during the regeneration of Parkin^−/−^ muscles. The results show that Parkin^−/−^ muscle have lower Tom20 levels in skeletal muscle at baseline compared to WT muscle (Figure 6D). However, we observed a significant increase in Tom20 protein levels in Parkin^−/−^ muscles 21dpi compared to WT (Figure 6D). These results suggest that Tom20 might be necessary for late myofibre/myotube assembly in both in vivo and in vitro models, respectively.

To better understand the effects of Parkin on mitochondrial dynamics, we analyzed DRP1 and MFN2 protein levels. Our results demonstrated a significant increase in DRP1 in Parkin^–/–^ mice at 10dpi compared to WT mice (Figure 6E). We also observed an increase in VDAC2/DRP1 colocalization at myofibre 10dpi (Figure 6F). Levels of MFN2, a mitochondrial fusion protein, was not altered in the absence of Parkin during the regeneration (Figure 6G). On the other hand, immunofluorescence results indicated a lower co-localization between MFN2 and VDAC2 in myofibre of Parkin^−/−^ mice (arrow heads), and an increase of MFN2 labeling at MuSCs around the myofibre (arrows, Figure 6H). Taken together, our results suggest that DRP1 is increased in mitochondria during the myofibre maturation in Parkin^−/−^ mice, while MFN2 is reduced.

**Figure 7:**
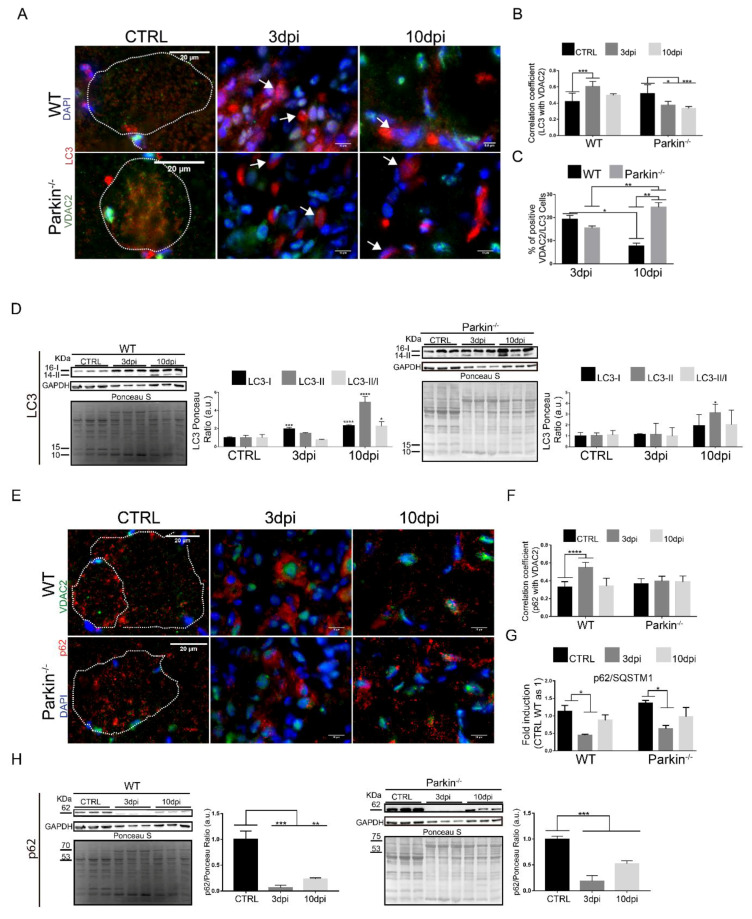
Absence of Parkin results in autophagy deregulation during skeletal muscle regeneration. (**A**) Immunofluorescence of LC3 (red), VDAC2 (green), and DAPI (blue) in TA muscle cryosections from WT and Parkin^–/–^ mice at 3 and 10 dpi. The myofibers are outlined by dotted area. The images were acquired with a 40× objective. Bars represent 20 µm in CTRL, 10 µm in 3dpi and 10dpi. (**B**) Analysis of VDAC2 and LC3 colocalization using Pearson’s Correlation Coefficient. Data represent means ± SD (One-way ANOVA: * *p* < 0.05; *** *p* < 0.01; *n* = 5). (**C**)% of VDAC2/LC3 positive cells. Data represent means ± SD (One-way ANOVA: * and ** *p* < 0.05; *n* = 5). (**D**) Western blot and densitometry analysis of LC3 during the regenerative process in TA muscle from WT and Parkin^–/–^ mice in CTRL and at 3 and 10dpi; comparisons were made with the TA muscle in CTRL of each genotype. Ponceau S staining was used to normalize for loading. GAPDH was modulated by injury and Ponceau was used as loading control. Data represent means ± SEM (One-way ANOVA: * *p* < 0.05; *** *p* < 0.01; **** *p* < 0.001 *n* = 5). (**E**) Immunofluorescence of p62 (red), VDAC2 (green), and nuclei I (blue) in TA muscle in WT and Parkin^–/–^ mice in CTRL and at 3 and 10dpi. The dotted areas outline myofibers. The images were acquired with a 40× objective. Bars represent 20 µm in CTRL, 10 µm in 3dpi and 10dpi. (**F**) Analysis of the co-localization between VDAC2 and p62 using Pearson’s Correlation Coefficient. Data represent means ± SD (One-way ANOVA: **** *p* < 0.0001; *n* = 5). (**G**) p62/SQTM1 relative mRNA expression (RT-PCR) in TA muscle from WT and Parkin^–/–^ mice at 3dpi and 10dpi compared with CTRL (CTRL WT as 1). Data represent means ± SD (One-way ANOVA: * *p* < 0.05; *n* = 5). (**H**) Western blot analysis of p62 during the regenerative process in TA muscle from WT and Parkin^–/–^ mice in CTRL and at 3 and 10dpi; comparisons were made with the TA muscle in CTRL of each genotype. GAPDH was modulated by injury procedure and Ponceau was used as loading control. Data represent means ± SEM (One-way ANOVA: ** *p* < 0.05; *** *p* < 0.01; *n* = 5).

To understand the effects of Parkin deficiency on mitophagy during regeneration, we analyzed autophagy proteins LC3 and p62 by immunofluorescence and western blot in control and injured muscle. Our results showed reduced co-localization between LC3-positive autophagosomes and mitochondrial VDAC2 at 3 and 10dpi in muscles of Parkin^−/−^ mice (Figure 7A,B). Interestingly, we found elevated LC3 and VDAC2 positive cells in Parkin^−/−^ mice at 10dpi (Figure 7C). 

Our Western Blot results also indicated differential regulation of LC3-I/LC3-II conversion during the regeneration in Parkin^−/−^ mice (Figure 7D). We also noted that Parkin^–/–^ mice showed no increase in p62 and VDAC2 co-localization at 3dpi compared to in WT mice (Figure 7E,F). However, the p62 levels were not significantly altered in Parkin^−/−^ mice (Figure 7G,H). Overall, these data indicate that recruitment of autophagic proteins to mitochondria can occur even in absence of Parkin during the regeneration.

## 3. Discussion

Myogenic precursor cells, also called satellite cells, are key factors in maintaining homeostasis of adult skeletal muscle tissue. Defects in pathways involved in maintaining the quiescent state or regulating the differentiation process of these cells can lead to development of sarcopenia, as observed in aging or myopathy-related conditions [17,18]. After injury of muscle, some of the cells will remain as quiescent progenitor cells to maintain the pool of satellite cells in the tissue while others are activated. The activated cells start to proliferate and differentiate to regenerate the muscle tissue [19]. Recently, mitophagy was reported to be o required for the removal of uncoupled mitochondria during C2C12 cells differentiation [8]. However, the role of Parkin in the differentiation process in primary cells and tissue is still unclear. The most important findings in our study are: MuSCs express Parkin and its deficiency in this protein leads to altered MuSCs differentiation due to defects in myogenic program, mitophagy dysregulation and changes in mitochondrial proteins profile expression.

Parkin has been described as a critical factor for mitochondrial quality control and loss-of-function mutations in Parkin are known to lead to early onset Parkinson’s disease [20,21]. Studies have also focused on Parkin’s function in skeletal muscle as myofibers have very high mitochondrial content [22]. In addition, some patients with Parkinson disease develop myopathy [23,24]. It has also been demonstrated that Parkin is crucial for maintaining normal mitochondrial function and contractile activity in adult/mature myofibers [25]. Here, we observed that Parkin is expressed in skeletal muscle fibers, myoblasts, and myotubes in culture and in MuSCs during the regeneration process (3dpi peak). Peker et al. demonstrated that Parkin is critical for proper size of myotubes and suggested that Parkin participates in muscle growth and development [14]. Indeed, we detected smaller cross-sectional area of myofibers in Parkin knockout mice compared with wild-type mice in non-injured muscles. Moreover, myofiber size during the assembly process or mature fibers post-injury were consistently reduced, demonstrating the relevance of Parkin to myofiber size and recovery of muscle mass after injury. A critical discovery in our study was the histological changes in tissue structure at 21 days of regeneration, thus indicating that those effects precede a potential myopathy condition.

In our study, we hypothesized that Parkin modulates myogenic factors, influencing the levels of differentiation of myofibre and consequently the size of mature myofibers. The full recovery of myofibers and muscle mass after injury is dependent on the capacity of the MuSCs to proliferate and differentiate after injury. Several factors have been described to be involved in this orderly process [26]. Pax7 is one of the most crucial factors in satellite cells which ensures that cells retain their proliferative potential while inhibiting differentiation [1]. On the other hand, Myogenin, a late phase myogenic factor, is expressed in MuSCs that are committed to fusion and regeneration of myofibreor to formation of new myotubes [27]. In our study, we observed an increase in Pax7 and robust reduction in Myogenin in Parkin^−/−^ skeletal muscle, suggesting that the MuSCs are more committed to proliferation than differentiation. Importantly, high levels of Pax7 and reduced mitochondrial density has been suggested as markers for lower myogenic commitment [28,29]. Moreover, studies have reported that a consequence of reduced mitophagy is decreased Myogenin levels [7,30]. We observed an accumulation of Cyclin D1 and Myh3 in Parkin^−/−^ mice during the regenerative process, reinforcing the idea of incomplete differentiation process when mitophagy is impaired. Taken together, our results suggest that MuSCs can undergo differentiation in the absence of Parkin, though it is delayed and might maintain the cells in a “myoblast/myocyte-proliferative” state.

Recently, mitophagy was identified to be a crucial mechanism underlying myoblast differentiation. Sin et al. demonstrated the importance of functional mitophagy for differentiation of [8]. In this study, the authors demonstrated that a metabolic change, which is dependent on mitochondrial removal, is important for the assembly of myofibre. In addition, mitophagy has been implicated in reduction of oxidative stress during regeneration/differentiation processes [30,31]. Therefore, we examined mitochondrial proteins and markers of autophagic flux in Parkin^–/–^ mice during skeletal muscle regeneration. We used Tom20 and VDAC2 as mitochondrial markers since there was a significant differential modulation of both proteins in the C2C12 differentiation process. WT mice and Parkin^–/–^ mice had reduced VDAC2 levels after injury; however, in the absence of Parkin, we did not observe the recovery of VDAC2 protein levels. VDAC2 is a voltage channel highly expressed in the skeletal muscle, and its expression is necessary for the maintenance of mitochondrial quality [32]. Here, we observed increased levels of Tom20 at 21dpi in Parkin^−/−^ mice, indicating the presence of a new mitochondrial population. Thus, these results suggest the presence of a mitochondrial population with potentially reduced voltage control in Parkin^−/−^ mice. This observation could explain the increased DRP1 and reduced MFN2 observed in these mice, pointing to a more fragmented mitochondrial network during muscle regeneration in Parkin^−/−^ mice.

Mitophagy is closely linked to mitochondrial biogenesis [33]. Ivankovic et al. reported coordinated mitochondrial and lysosomal biogenesis following activation of PINK1/Parkin-mediated mitophagy in the neuroblastoma SH-SY5Y cell line [33]. In addition, evidence suggests that Parkin forms an important Parkin-PARIS-PGC-1 axis controlling signaling for mitochondrial biogenesis during muscle stress [34]. Indeed, we suggest that, in absence of Parkin, there is a delay in the mitophagic process that could lead to a delayed mitochondrial biogenesis during the regeneration. Moreover, Parkin overexpression has been demonstrated to improve mitochondrial function and to protect cells from proteotoxicity, suggesting that selective elimination of damaged mitochondria enhances cellular homeostasis [35]. Hence, our results indicated a possible mixed mitochondrial population in Parkin^−/−^ mice, with non-removed (VDAC2-/Tom20+) and new synthesized (VDAC+/Tom20+) mitochondria. This mixture might affect the function of MuSCs at several steps of the differentiation process, resulting in delayed regeneration and reduced capacity to metabolic changes adjustment.

To our knowledge, this is the first study identifying the importance of Parkin in MuSCs homeostasis and in skeletal muscle regeneration. Parkin is an important regulator of mitophagy and safeguards the quality of the mitochondrial network. Our results suggest that Parkin plays a key role in mitochondrial protein levels of satellite cells during the regeneration process. We demonstrated that the presence of Parkin is critical for regulation of myogenic factor levels, mitochondrial dynamics, and autophagic flux during regeneration. 

We believe that our study provides increased insights into the capacity of MuSCs to adapt to enhanced metabolic demand during the differentiation and increased knowledge can help to clarify myopathies and fatigue signs observed in Parkinson’s disease patients. We propose that mitochondrial quality control as a key factor in skeletal muscle recovery.

## 4. Materials and Methods

### 4.1. Animals

C57BL/6J wild type (WT) and Parkin knockout male mice (10-12 weeks old, 20–26 g, N = 35 for each genotype) were used in these studies. The Parkin^tm^1Shn (Parkin^−/−)^ mice were generated by [36], provided by the Multidisciplinary Center for Biological Research (CEMIB) and transported to the School of Applied Sciences of the University of Campinas in Limeira (São Paulo, Brazil). All animal experiments were approved by the Ethics Committee on the Use of Animals of the University of Campinas (CEUA, protocol no. 4687-1/2017 approved in August, 31th 2017). The mice animals were submitted to surgical procedure to expose the TA muscle and the injury was induced by injection of cardiotoxin in the TA muscle of the posterior left paw. The mice were pre-anesthetized with analgesic (buprenorphine—0.1 mg/kg) and anesthetized (ketamine—80 mg/kg; and xylazine—10 mg/kg), the left tibialis anterior (TA) muscle exposed and CTX from *Najamossambicamossambica* (C9759, Sigma-Aldrich^®^, Saint Louis, MO, USA) in PBS was injected (20 μL of 10 μM) at four longitudinally sites. The mice were harvested at 3dpi, 10 10dpi, and 21dpi. The TA muscle from the posterior right paw of all mice was used as the control group (CTRL) in each group. After euthanasia, the TA muscle was removed, weighed, divided and frozen in isopentane or liquid nitrogen.

### 4.2. Cell Culture

Mouse skeletal muscle myoblast C2C12cells (CRL-1772™, ATCC^®^, Manassas, VA, USA) were cultured in Dulbecco’s Modified Eagle Medium (DMEM—D5796, Sigma-Aldrich^®^) supplemented with 10% bovine serum (F2561, Sigma-Aldrich^®^, Saint Louis, MO, USA), antibiotics (15140122, Gibco^®^ Thermo Scientific^®^, Carlsbad, CA, USA), and glutamine (604690, Sigma-Aldrich^®^, Saint Louis, MO, USA) for 48 h until reaching 60% confluence. The growth medium was removed, cells were washed twice with phosphatase buffer saline (PBS) and then trypsinized (25200-072, Gibco^®^ Thermo Scientific^®^, Carlsbad, CA, USA). Cell differentiation was induced by incubating cells in DMEM medium containing 2% horse serum (16050-122, Gibco^®^ Thermo Scientific^®^, Carlsbad, CA, USA), antibiotics, and glutamine for up to 2, 4, and 6 days. The control cells (CTRL group) were used as undifferentiated cells. For cell culture, we used triplicates for each treatment, and replicated the independent experiment three times.

### 4.3. Mitochondrial Fractionation

Mitochondrial isolation from cells was performed using the cell culture mitochondrial extraction kit (Sigma-Aldrich^®^, Saint Louis, MO, USA) according to the manufacturer’s instructions. Briefly, were homogenized using a Dounce homogenizer and mitochondrial fraction isolated using differential centrifugation (700× *g* for 10 min; 12,000× *g* for 15 min; 3000× *g* for 15 min, at 4 °C). After the final centrifugation, the supernatant was transferred to a new microtube, which represents the cytosolic fraction of mitochondria. The mitochondrial pellet was washed by and centrifugation at 12,000× *g* at 4 °C for 5 min. The supernatant was discarded and the remaining pellet represents the fraction of mitochondria.

### 4.4. Mophological Analyzes

Frozen anterior tibial muscles were cut into 10μm cross-sections from their mid-part, using a cryostat (CM1850, Leica^®^, Buffalo Grove, IL, USA) at −24 °C and adhered to slides in serial cuts. Subsequently, muscles were stained using hematoxylin-eosin (H&E) and modified by Gomoritrichrome staining. The images were acquired using a light microscope (Leica^®^). The cross sectional area was measured using ImageJ software (Research Services Branch, National Institute of Mental Health, Bethesda, MD, USA).

Cryosections were performed in serial sections and images for immunofluorescence and hematoxylin and eosin analyzes were acquired in each section to obtain representative analysis of the TA muscle. In immunofluorescence experiments, the exposure time was controlled according to the objective used and the background signal of the negative control used by adjusting the best signal with the least background. In general, a 40× objective used between 1 to 1.5 s of exposure time and a 100× objective used between 300 to 600 ms.

The correlation analyses of immunofluorescence experiments were performed by the Pearson’s Correlation Coefficient using the Icy software and the Colocalization Studio plugin according to [37]. In these analyzes, 4-6 images of different regions (and sections) of the TA muscle per mouse were used. This colocalization was confirmed by Van Steensel’s test. In the analysis of positive cells for Pax7 and Myogenin, between 800 to 1000 cells per mouse were counted. The analysis of Myh3 positive cells was done by counting positive cells from 4 TA muscle images per mouse and in the measurement of the total area of Myh3, the plugin HK-means from the Icy software was used. For the quantification of CSA, the software ImageJ was used, measuring between 800 to 1000 myofibres per mouse present in four images of different regions (and sections) of the TA muscle.

### 4.5. Immunofluorescence

Slides with cryosections were washed with PBS for5 min and fixed with 4% Paraformaldehyde (PFA) in PBS for 10 min, then washed 3 times for 15 min with PBS containing 0.1% of Tween 20 (PBS-T), post-fixed with cold methanol at 4 °C for15 min, and washed in PBS-T. The slides were immersed in a Citrate buffer at 100 °C for 15 min and washed with PBS-T for 15 min changing the solution each 5 min. Next, slides were incubated in pre-blocking solution (PBS-T, maleic acid and 3% bovine serum albumin—BSA) for 1 h in a wet chamber at room temperature. Slides were washed with PBS-T and the blocking solution was added (PBS-T, 3% BSA, and 1% glycine) with the antibodies, incubed in a wet chamber and at 4 °C overnight. The slides were washed with PBS-T, the secondary antibody solution was incubated for 1 h in a dark and wet chamber at room temperature. The slides were briefly washed with PBS-T and exposed to a Sudan Black B solution (0.3% Sudan Black, 70% ethanol) for 1.5 min. Slides were briefly washed in 50% ethanol and proceeding to 4 washes of 5 min with PBS. Finally, mounting medium with DAPI (VECTASHIELD^®^, Vector Laboratories, Burlingame, CA, USA) was added and sealed with coverslips.

For immunofluorescence of C2C12 cells, the cells were plated under a coverslip (previously immersed in a 6M chloride acid solution, overnight, inside a 24-well cell culture plate). Cells were fixed with 4% PFA in PBS for 10 min and permeabilized in PBS-T. Subsequently, they were incubated in blocking solution of PBS-T containing 3% BSA and 1% glycine for 1 h at room temperature, and then incubated with primary antibodies in blocking solution (PBS-T, 3% BSA and 1% glycine) overnight, at 4 ºC (see Supplementary Table S1). After three washes with PBS-T, the cells were incubated with secondary antibodies in blocking solution of 1% BSA in PBS-T for 1 h in a dark room.

### 4.6. Confocal Images

The images were acquired using a LSM-780-NLO confocal microscope (Carl Zeiss MicroImaging GmbH, Jena, Germany) at the Core Facility for Scientific Research—University of São Paulo (USP) (CEFAP-USP).

### 4.7. Western Blot

The tissue and cells were homogenized in extraction buffer (0.625% Nonidet P-40, 0.625% [*w*/*v*] sodium deoxycholate; 0.00625M sodium phosphate pH 7.2, 1mM EDTA pH 8 and 1% phosphatase inhibitor (#P2850, Sigma-Aldrich^®^, Saint Louis, MO, USA) and protease inhibitor (#P8340, Sigma-Aldrich^®^, Saint Louis, MO, USA) and centrifuged at 15,000× *g* for 10 min. Forty µg of proteins were applied to electrophoresis in SDS—PAGE gel prepared between 12–14%. The proteins were transferred to a nitrocellulose membrane and then stained with Ponceau Red (Sigma-Aldrich^®^). After blocking with solution of Tris buffer saline with 0.1% Tween 20 (TBS-T) and 5% of milk, the primary antibodies (see Supplementary Table S1) were incubated in blocking solution of 5% of BSA in TBS-T, overnight, at 4 °C. The membranes were washed in TBS-T and incubated with secondary antibodies (peroxidase-conjugated antibody; see Supplementary Table S1) for one hour at room temperature. The images were acquired using Pierce™ ECL Western Blotting Substrate (32106, ThermoFisher Scientific^®^, Carlsbad, CA, USA) and printed using G:BOXChemi XRQ Photodocumentation powered by GeneSys software(SYNGENE^®^, Frederick, MD,) or Carestream® BioMax® MR film (Z350397, Sigma-Aldrich^®^, Saint Louis, MO, USA) to detect the chemiluminescence signal.

### 4.8. Real-Time PCR

To extract total RNA, the TA muscle was homogenized in 1ml of Trizol (Invitrogen, Carlsbad, CA, USA), following the manufacturer’s recommendations. Purity and quantitation were assessed by spectrophotometry (Epoch BioTek^®^, Winooski, VT, USA). Reverse transcriptase reaction was performed using 1 ug of extracted RNA, OligodT (500 ug/mL), 10 nM dNTP Mix, 5× First Strand Buffer, 0.1M DTT, and 200U reverse transcriptase (MMLV Reverse Transcriptase—Promega Corporation, Madison, WI, USA). Gene expression was analyzed by fluorescence quantification with QuantStudio Real-Time System (ThermoFisherScientific^®^, Carlsbad, CA, USA). The PCR reaction was performed with complementary DNA (cDNA), in a Mix containing Power SYBR Green PCR Master Mix (Applied Biosystems, ThermoFisherScientific^®^, Carlsbad, CA, USA), ultrapure water, specific primers (See Supplementary Table S2). Gene expression was analyzed by the formula: 2-DDCt, described by K. Livak in the Biosystem Sequence Detector Bulletin 2.

### 4.9. Statistical Analyses

Densitometry analyses were performed with ImageJ software. To verify the distribution of the data we used the Shapiro-Wilk normality test and Brown-Forsythe test to compare the variances. The data were compared between the groups using One-way and Two-way ANOVA with Bonferroni’s multiple comparisons and Student’s *t*-test (GraphPadPrism 7 software, Graphpad, San Diego, CA, USA). The level of significance was set at *p* < 0.05. Data are presented with the respective means and Standard Deviation or Standard Error values.

## Supplementary Materials

Supplementary materials can be found at https://www.mdpi.com/1422-0067/21/21/8007/s1. Figure S1, Western blot for control proteins, immunofluorescence for negative control and cell culture open field images; Figure S2, Immunostaining of Pax7 and Myogenin; Figure S3 Immunostaining of NCAM during regeneration; Figure S4, Western blot for VDAC2; Table S1, Primary and Secondary Antibodies used in Immunofluorescence and Western Blot; Table S2 Primers Sequence.

## Abbreviations

CSA	Cross-sectional area
CTRL	Control
CTX	Cardiotoxin
Dpi	Day post-injury
DRP1	Dynamin-related protein 1
Gas	Gastrocnemius
LC3-I	Microtubule-associated protein 1A/1B-light chain 3-I
LC3-II	Microtubule-associated protein 1A/1B-light chain 3-II
MFN2	Mitofusin 2
MuSCs	Muscle stem cells
Myh3	Myosin heavy chain 3
NCAM	Neural cell adhesion molecule
p62/SQSTM1	Sequestosome 1
Pax7	Paired-box transcription factor 7
TA	Tibialis Anterior
Tom20	Translocase of outer membrane 20
VDAC2	Voltage dependent anion-selective channels 2
