# Supplementary material for: Loss of Parkin Results in Altered Muscle Stem Cell Differentiation during Regeneration
Source: Int J Mol Sci. 2020 Oct 28;21(21):8007. doi: 10.3390/ijms21218007 (PMC7662548; doi:10.3390/ijms21218007)
Supplement: Supplementary file 1 [file ijms-21-08007-s001.pdf]

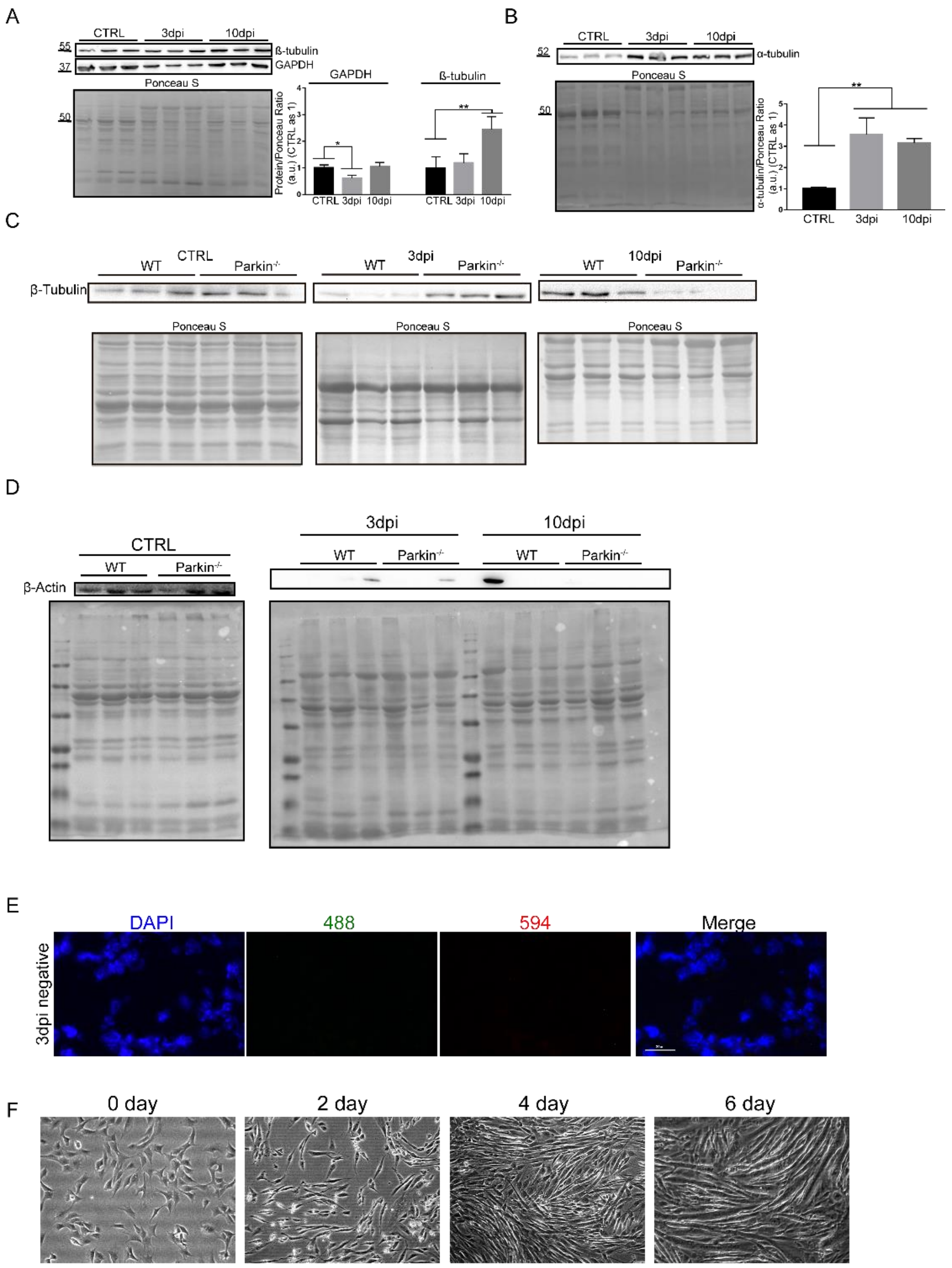

Figure S1. Western Blotting internal control experiments. **(A)** Western Blot of β-Tubulin and GAPDH in TA muscle from WT mice in CTRL and at 3 and 10dpi. Data represent means ± SD (t-test: * $p < 0.05$; ** $p < 0.01$; $n = 3$) **(B)** Western Blot of α-Tubulin in TA muscle from WT mice in CTRL and at 3 and 10dpi. **(C)** Comparison of protein levels of β-Tubulin in Parkin$^{-/-}$ mice to WT in CTRL, 3dpi and 10dpi. **(D)** Comparison of protein levels of β-Actin in Parkin$^{-/-}$ mice to WT in CTRL, 3dpi and 10dpi **(E)** Negative control experiments related to Figure 1 D. **(F)** Optical images of Myoblasts C2C12 during differentiation related to the experiments in Figure 2.

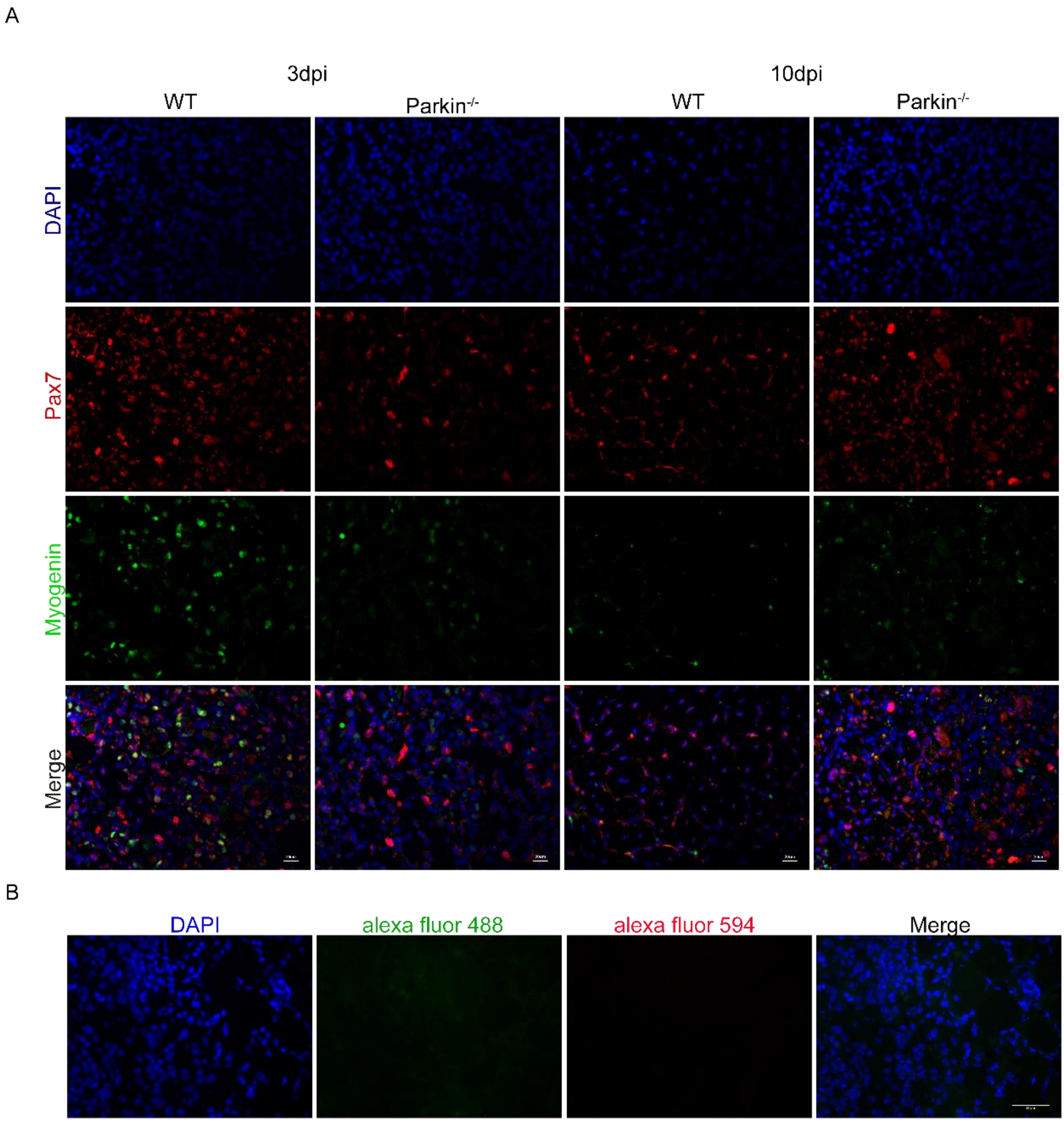

Figure S*2* Immunostaining of Pax7 and Myogenin. **(A)** Split channel images of figure 5. **(B)** Negative control experiments related to Figure 5 A.

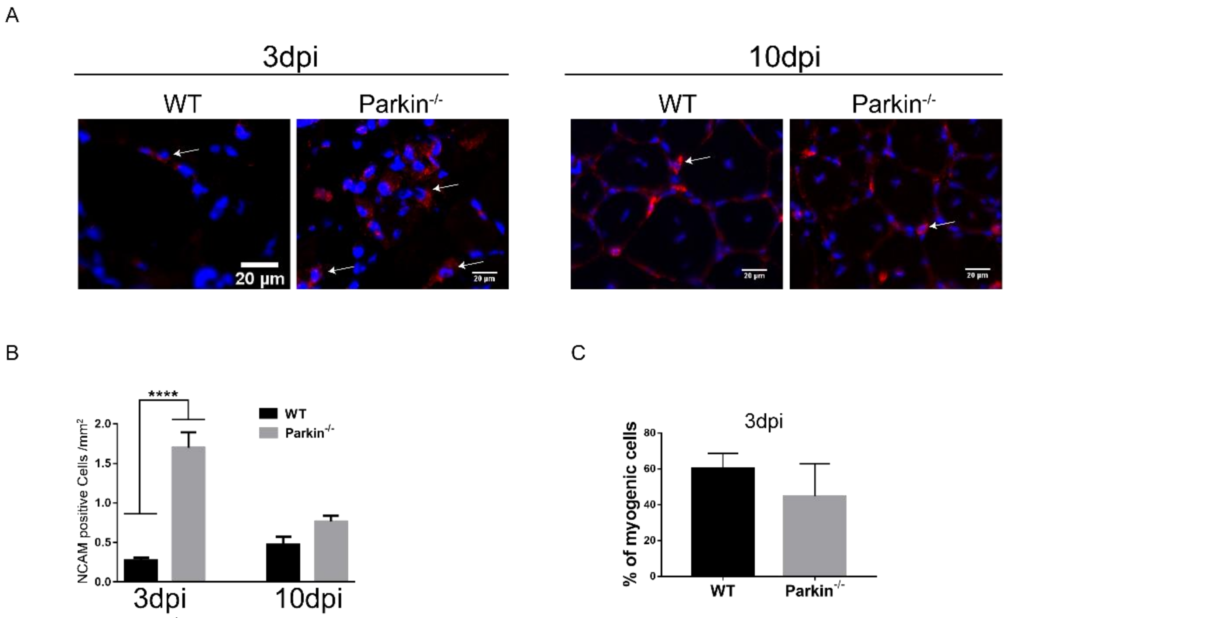

Figure S3. Parkin$^{-/-}$ mice have increased NCAM^{+} cells in TA muscle at 3 days post-injury. **(A)** Immunofluorescence of NCAM (red) and DAPI (blue) in TA muscle from WT and Parkin$^{-/-}$ mice at 3 and 10dpi. White arrows indicate NCAM^{+} cells. **(B)** Quantitation of NCAM positive Cells/mm^2. Data represent means ± SD (t-test: **** $p < 0.0001$; n = 4). **(C)** % of myogenic cells (Pax7 + Myogenin Cells) at 3dpi from WT and Parkin mice. Data represent means ± SD.

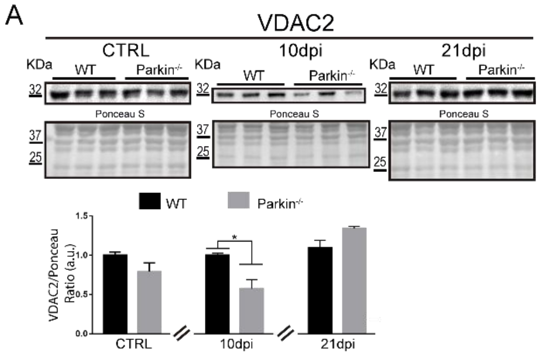

Figure S4 Decreased VDAC2 expression in Parkin$^{-/-}$ mice at 10 days after injury. **(A)** Western Blot and densitometry analysis of VDAC2 levels during the regenerative process in CTRL, 10dpi and 21dpi TA muscle from WT and Parkin$^{-/-}$ mice. Ponceau S staining was used to normalize for loading. Data represent means ± SEM (t-test: * $p < 0.05$; n = 5)

Supplementary tables 1

	Primary Antibody	Brand	ID	Proportion
Immunofluorescence	Anti-Myogenin	Abcam	ab103924	1:200
	Anti-Parkin	Abcam	ab15494	1:100
	Anti-Parkin [PRK8]	Abcam	ab77924	1:250
	Anti-Pax7	Abcam	ab34360	1:250
	Anti-SQSTM1/p62	Abcam	ab91526	1:300
	Anti-VDAC2	Abcam	ab37985	1:250
	Anti-LC3A/B	Cell Signaling	#4108S	1:300
	Anti-NCAM	Cell Signaling	#3606	1:300
	Anti-MYH3	Origene	TA349138	1:500
	Anti-MFN2	Abcam	ab124773	1:250
Western Blotting	Anti-SQSTM1/p62	Abcam	ab91526	1:2000
	Anti-Myogenin	Abcam	ab103924	1:2000
	Anti-Parkin [PRK8]	Abcam	ab77924	1:1000
	Anti-Pax7	Abcam	ab34360	1:1000
	Anti-VDAC2	Abcam	ab37985	1:1000
	Anti-α-tubulin	Cell Signaling	#2125s	1:3000
	Anti-β-tubulin	Cell Signaling	2146s	1:3000
	Anti-Cyclin D1	Cell Signaling	#2978s	1:2000
	Anti-Parkin	Cell Signaling	#2132S	1:1000
	Anti-LC3A/B	Cell Signaling	#4108S	1:2000
	Anti-GAPDH	Thermo	PA1-9871-5	1:5000
	Anti-MFN2	Abcam	ab124773	1:1000

Second Antibody	Brand	ID	Proportion
Alexa Fluor® 594 AffiniPure Donkey Anti-Rabbit IgG	Jackson	711-585-152	1:250
Alexa Fluor® 488 AffiniPure Donkey Anti-Goat IgG	Jackson	705-545-003	1:250
Alexa Fluor® 488 AffiniPure Donkey Anti-Mouse IgG	Jackson	715-545-1150	1:250
Donkey Anti-Goat Polyclonal	Jackson	705-035-003	1:10000
Goat Anti-Rabbit Polyclonal	Jackson	111-035-003	1:10000

Supplementary table 2

Primers Sequence		
Gene	Foward	Reverse
Park2	TGTCCCAACTCCCTGATTAAAG	ACAGCACACCTCCCATTTG
p62/SQSTM1	GAACTCCAGTCTCTACAGAT	CGATGTCGTAATTCTTGGTC
18s	GCAATTATTCCCCATGAACG	GGCCTCACTAAACCATCCAA
